# Study on Restraint Effect of Post-Casting Belt in Full-Section Interval Casting Immersed Tube

**DOI:** 10.3390/ma18204665

**Published:** 2025-10-10

**Authors:** Bang-Yan Liang, Wen-Huo Sun, Yong-Hui Huang, Kai Wang

**Affiliations:** 1 The Second Engineering Company of CCCC Fourth Harbor Engineering Co., Ltd., Guangzhou 510230, China; liangbangyan@ccccltd.cn (B.-Y.L.); sunwenhuo@ccccltd.cn (W.-H.S.); 2 Research Center of Wind Engineering and Engineering Vibration, Guangzhou University, Guangzhou 510006, China; huangyh@gzhu.edu.cn

**Keywords:** integral immersed tube tunnel, full-section casting, post-casting belt, restraint effect, concrete crack control

## Abstract

The Chebei integral Immersed Tunnel introduced an innovative full-section interval casting process, where post-casting belts impose restraint effects on the full-section casting segments. To mitigate concrete cracking, this study investigates the influence of the bottom steel plate and steel bars in the post-casting belts on the mechanical behavior of full-section casting segments through comparative analysis of field tests and numerical simulations. Requirements for post-casting belt length are proposed. Key findings include: under post-casting belt restraint, the full-section casting segment’s shrinkage strain reached 348 με, with hydration heat-induced cooling and drying shrinkage contributing 60% and 40%, respectively. A temperature-dependent thermal expansion coefficient model was developed to characterize the nonlinear relationship between concrete strain and hydration heat temperature. Restraint effects diminished with increasing post-casting belt length, and the post-casting belt length should be control. At 1.6 m (Chebei design), restraint-induced tensile stress was 1.4 MPa (restraint coefficient β = 0.12), with the bottom steel plate and steel bars contributing about 70% and 30%, respectively. Relationships between post-casting belt length, stress, and restraint coefficient are established for engineering reference. These research findings have been successfully applied in the Chebei Immersed Tunnel, enabling high-quality prefabrication of full-section interval casting immersed tubes.

## 1. Introduction

Immersed tunnel technology has become essential for cross-river/sea corridors due to its adaptability to complex hydrogeological conditions. According to the International Tunnelling Association (ITA) 2023 report, China contributed 63% of global immersed tunnels constructed in the last five years. While the Hong Kong–Zhuhai–Macao Bridge [1,2] and Dalian Bay tunnels [3] used segmental designs, integral structures dominate recent projects [4,5,6,7] (e.g., Chebei Tunnel, Xiangyang Tunnel, Huizhanxi Crossing Tunnel).

Watertightness is critical for operational safety and durability. Concrete self-waterproofing is fundamental, yet thermal cracking remains a primary threat [8,9,10]. The formation of concrete cracks primarily stems from the synergistic interaction of two core reasons: significant heat release during cement hydration creates substantial temperature differentials between the interior and exterior of the structure, generating thermal stresses [11,12,13,14]. Volume shrinkage during cooling and drying phases induces restraint stresses when constrained by the structure itself or external conditions. Cracks occur when these stresses exceed the concrete’s tensile strength at the corresponding age, severely compromising waterproofing performance and long-term durability. The essence of restraining stress control lies in resolving the conflict between shrinkage deformation and external constraints.

**Figure 1:**
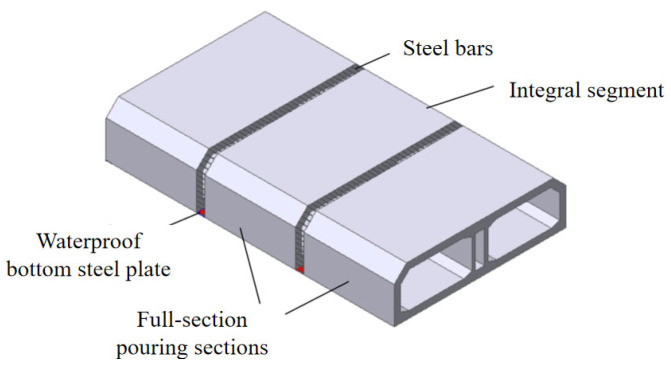
Full-section interval casting diagram of the Chebei Immersed Tunnel [18].

Current monolithic immersed tubes often employ “post-casting belts + layered casting” [15,16,17,18]. Although post-casting belts partially relieve shrinkage stress, construction joints between layers create vulnerable zones for crack initiation. The Hong Kong–Zhuhai–Macao Bridge pioneered segmental full-section casting, reducing joints and cracking rates to <5% [19]. Building on this, Chebei Tunnel innovated a “full-section casting + post-casting belts” process for integral tubes to eliminate interlayer joints. And this process is referred to as full-section interval casting in this article. The integral immersed tube consists of multiple segments connected by post-casting belts, allowing continuous steel bars and featuring a waterproof steel plate that spans the entire tube. Both the segments and the post-casting belts employ full-section casting technology, as shown in Figure 1. The Chebei Tunnel is a 123 m long integral immersed tube, divided into seven 16.2 m long full-section casting segments and six 1.6 m long post-poured belts.

However, this process led to a new issue: continuous waterproof steel plates and steel bars across post-casting belts introduce restraint forces on the full-section casting segment, creating cracking risks. For the concrete crack control for the integral immersed tube of Chebei Tunnel, the core problem that needs to be solved is to control the influence of post-casting belt constraints on concrete stress. Therefore, this article grasps the volume shrinkage law of the full-section casting segment under the constraint of the post-casting belt through on-site testing and numerical calculation. It is necessary to reduce the constraint effect and prevent concrete cracking.

## 2. Field Test Analysis

### 2.1. Instrumentation

**Figure 2:**
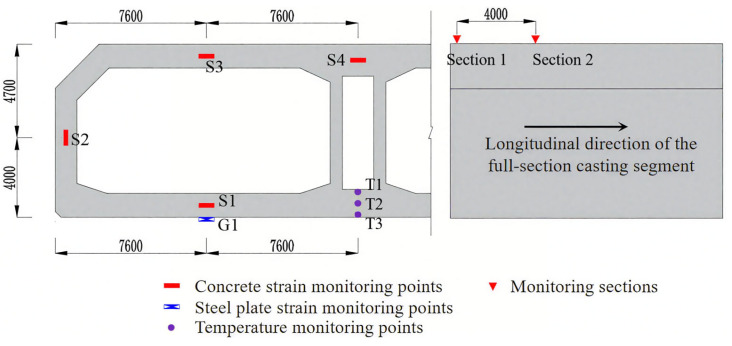
The layout diagram of measurement points [18].

Two monitoring sections (designated as Section 1 and Section 2) were positioned at the termination of the full-section casting segment, located 0.2 m and 4.2 m from the post-casting belt, respectively. Four strain measurement points (S1–S4) were positioned at the center of each section, with vibrating string non-stress gauges (BGK4200, Geokang Technologies Co., Ltd., Beijing, China) employed to measure concrete strain. A vibrating string strain gauge (G1) was installed on the bottom steel plate directly below S1 at Section 1 to measure steel strain. Three temperature sensors (BGK3700, Geokang Technologies Co., Ltd., Beijing, China) (T1–T3) were embedded in the base to monitor concrete hydration heat temperature at Section 1, which are arranged at the center and surface positions, respectively. Figure 2 illustrates the measurement point layout and field installation configuration.

### 2.2. Field Test Data Analysis

To reduce the risk of concrete cracking, the Chebei project uses low heat and low shrinkage concrete for the immersed tube, with a mix ratio shown in Table 1. And the curing temperature shall be not less than 35 °C, with humidity not less than 85%. Before formwork removal, adopt a curing method of covering with geotextile and sprinkling water for a duration of no less 3 days. After formwork removal, implement spray mist curing for a duration of no less 11 days.

**Figure 3:**
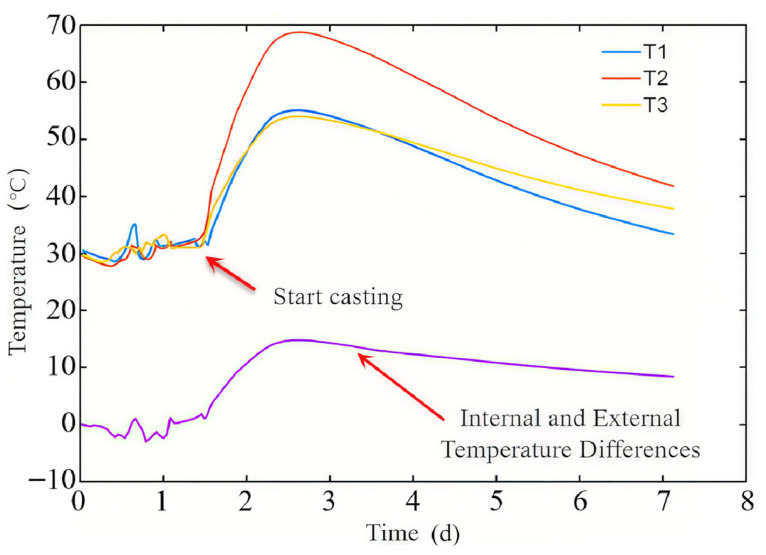
The variation diagram of hydration heat temperature over time.

Figure 3 shows the variation in hydration heat temperature over time. As shown in Figure 3, the temperature of concrete increases rapidly in the early stage of pouring, and reaches its maximum temperature about 1.5 days after pouring. At this time, the measured temperatures of the center and surface are 69.5 °C and 52 °C, respectively. The temperature decreases over time, reaching approximately 41.5 °C at the center and 35 °C on the surface after 5.5 days of pouring. The internal and external temperature difference first increases and then decreases with time, with a maximum temperature difference of about 15 °C.

The temperature of the section can be considered as a parabolic distribution [20], and the temperature at a certain point on the section can be calculated according to Formula (1), where *y* is the distance from the center, *h* is half of the plate thickness, and *T_core_*, *T_surf_* are the center temperature and surface temperature, respectively. And the plate thickness of the Chebei Tunnel project is 1.5 m.

(1) $$T\left(y\right)=T_{surf}+\left(T_{core}-T_{surf}\right)\cdot \left(1-\frac{y^{2}}{h^{2}}\right)$$

Then, the Formula (2) for calculating the average temperature of the cross-section can be obtained through Formula (1).

(2) $$T_{avg}=\frac{1}{2h}\int_{-h}^{h}T\left(y\right)dy=T_{surf}+\frac{2}{3}\left(T_{core}-T_{surf}\right)$$

After calculation, the average temperature of the bottom plate section is about 63.6 °C and 39.3 °C at 1.5 and 5.5 days after pouring, respectively.

**Figure 4:**
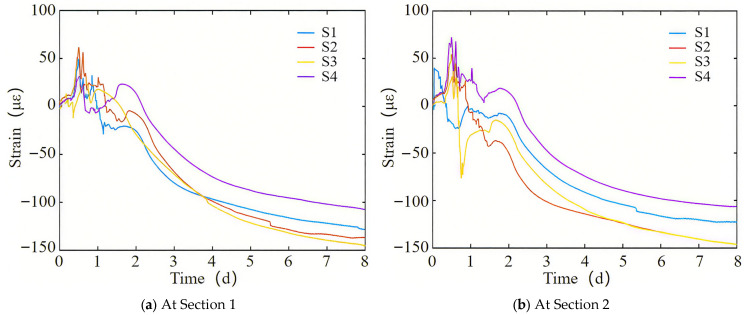
The measured strain variation diagram of the full-section casting segment with time.

Figure 4 shows the measured variation in strain over time of the full-section casting segment, with positive values indicating volume expansion and negative values indicating volume contraction. From Figure 4, it can be seen that the strain variation patterns of Section 1 and Section 2 are basically the same. The transverse strain of concrete in the early stage generally increases, with a maximum strain of about 60 με. As time progresses, the strain gradually decreases and stabilizes. After 8 days of concrete pouring, the transverse strains of the full-section casting segment can reach 145 με. The shrinkage strain of the bottom plate is smaller than that of the top plate.

The strain during concrete pouring can be divided into three parts: hydration heat temperature changes, self-drying shrinkage, and stress relaxation [21]. It can be calculated according to Formula (3), where *α_c_* is the thermal expansion coefficient of concrete, ∆*T* is the temperature difference, and *ε_dry_* and *ε_creep_* are the strains caused by dry shrinkage and stress relaxation, respectively.

(3) $$\varepsilon_{tot}=\alpha_{c}\cdot \Delta T+\varepsilon_{dry}+\varepsilon_{creep}$$

Due to the use of 85% humidity for maintenance in the Chebei Tunnel project, drying shrinkage and stress relaxation may not be considered in the early stage of concrete pouring. Test data shows that after 1.5 days of concrete pouring, the temperature increases from 30 °C to 63.6 °C, and the concrete strain increases by about 60 με. After 5.5 days of concrete pouring, the temperature decreases from 63.6 °C to 39.3 °C, and the concrete strain decreases by about 170 με. There is no linear relationship between concrete strain and temperature. Due to the increase in strength during the pouring process of concrete, it gradually hardens from the flow plastic state, and the thermal expansion coefficient of concrete also changes. This article proposes a calculation formula for the thermal expansion coefficient during the concrete pouring process through on-site testing and analysis, as shown in Formula (4), where $\alpha_{c0}$ a constant value with the value of 1.0 × 10^−5^, κ is the reduction factor, which can be taken according to Formula (5). This formula is obtained through analysis of on-site test data and can provide intuitive feedback on the variation in volume deformation with temperature during the development of concrete strength.

(4) $$\alpha_{c}=\kappa \alpha_{c0}$$

(5) $$\kappa =\left\{\begin{array}{cc} 0.15 & t\in \left(0,1.5d\right]\\ 0.7 & t\in \left(1.5d,5d\right]\\ 1 & t\in \left(5d,\infty \right) \end{array}\right.$$

**Figure 5:**
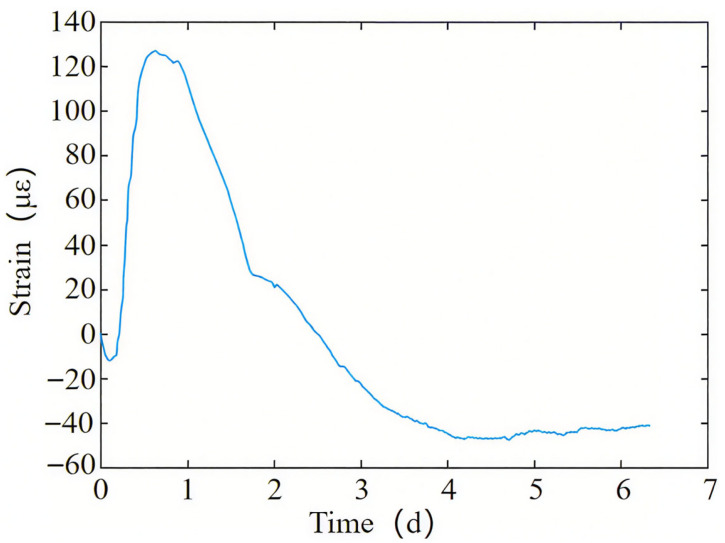
The measured strain variation diagram of the bottom steel plate over time.

Figure 5 shows the variation in measured strain over time of the bottom steel plate. It can be seen from Figure 5 that the steel plate undergoes corresponding expansion and contraction with the temperature rise and drop caused by the hydration heat of concrete. In the early stage of concrete pouring, the bottom steel plate expands rapidly in a lateral direction, with a maximum lateral deformation of 126 με. Subsequently, as the temperature decreases, the bottom steel plate gradually shrinks, and the overall bottom steel plate shows shrinkage. After 8 days of concrete pouring, the lateral shrinkage of the bottom steel plate is about 45 με.

Comparing Figure 4 and Figure 5, it is evident that during the initial stage of concrete pouring, the material is in a flow plastic state, and the bottom steel plate expands more than the concrete. As the concrete’s strength increases, the bottom steel plate and concrete integrate into a unified load-bearing structure and contract together.

## 3. Numerical Calculation Analysis

### 3.1. Finite Element Model

**Figure 6:**
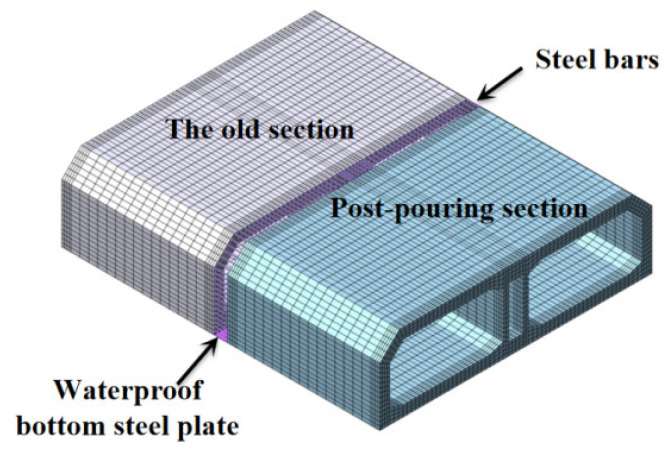
The FE model of the immersed tube.

As depicted in Figure 6, the full-section numerical model of the immersed tube is created using the finite element program MIDAS/FEA 2024. The post-casting belt is integrated with the surrounding immersed tube segments through steel bars. The immersed tube’s concrete is represented by three-dimensional solid elements with eight nodes and one-point Gauss integration. The steel plate at the bottom of the immersed tube is modeled using two-dimensional plate elements with four nodes and one-point Gauss integration, with a thickness of 6 mm. The primary element dimensions are approximately 0.2 to 0.4 m in the axial direction and 0.2 m in the thickness direction.

**Figure 7:**
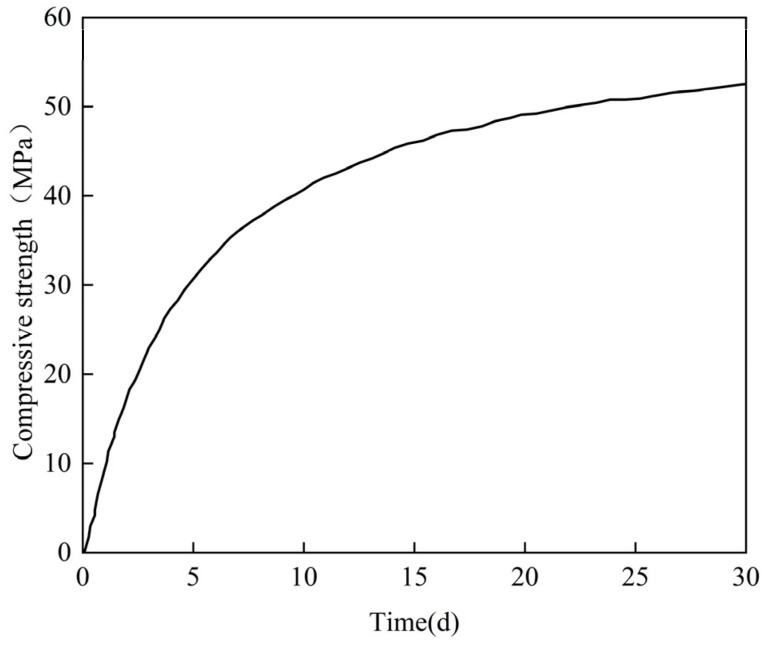
Curve of concrete compressive strength.

Parameters of concrete are given as follows, as per the literature [20]. The cube compressive strength after 28 days *f*_28_ is 52.5 MPa, the corresponding elastic modulus *E*_28_ is 35 GPa, the density is 2500 kg/m^3^, the thermal conductivity coefficient *m* is 9.54 k/(m·h∙°C), the specific heat capacity *c* is 0.973 k/(kg∙°C), and the thermal expansion coefficient *α*_c_ is taken according to Equation (4). Considering the continuous growth of concrete strength during the setting period, the change in concrete compressive strength is shown in Figure 7, based on Equation (6), where *f*_28_ is the compressive strength in 28 d; the parameter *a* is 4.5, and the parameter *b* is 0.95.

(6) $$f\left(t\right)=t_{eq}\cdot f_{28}/\left(a+b\cdot t_{eq}\right)$$

The process of analysis, the hydration reaction of mass concrete under adiabatic of conditions, the temperature *T(t)* increases with time *t* can be expressed by Formula (7), where *Q*_0_ is the highest adiabatic temperature rise in concrete, *W* is the cement dosage of unit volume concrete, *Q* is the hydration heat of cement, *k* is the reduction coefficient of hydration heat of admixture; *m* is the thermal conductivity coefficient of concrete.

(7) $$T\left(t\right)=Q_{0}\left(1-e^{-mt}\right)=k\frac{WQ}{\rho c}\left(1-e^{-mt}\right)$$

The CEB-FIP shrinkage [22] model is used for concrete, mainly considering factors such as environmental humidity, cement type, and concrete strength. The CEB-FIP shrinkage model can be expressed by Formulas (8)–(11), where *ε_sho_* is the coefficient of contraction effect, *β_s_* is the coefficient that describes the correlation between contraction and time, *β_sc_* is a constant, and its value depends on the type of cement used, *f_cm_* is the 28 day compressive strength of concrete, *β_RH_* is a constant, and its value is related to relative humidity; *A_c_* is the cross-sectional area of the component; *μ* is the circumference of the cross-section in contact with the atmosphere.

(8) $$\varepsilon_{d}\left(t,t_{c}\right)=\varepsilon_{sho}\beta_{s}\left(t-t_{c}\right)$$

(9) $$\varepsilon_{sho}=160+10\beta_{sc}\left(9-0.1f_{cm}\right)\times 10^{-6}\beta_{RH}$$

(10) $$\beta_{RH}=-1.55\left[1-{\left(\frac{RH}{100}\right)}^{3}\right]\;\;40\%\leq RH\leq 80\%$$

(11) $$\beta_{s}\left(t-t_{c}\right)=\sqrt{\frac{t-t_{c}}{350{\left(\frac{2A_{c}}{100\mu }\right)}^{2}+\left(t-t_{c}\right)}}$$

Parameters of steel are given as follows, as per the literature [23]. The elastic modulus *E*_s_ is 210 GPa, the density is 7850 kg/m^3^, the thermal conductivity is 180 kJ/(m·h·°C), the specific heat capacity is 0.46 kJ/(kg·°C), and the thermal expansion coefficient is 1.0 × 10^−5^.

### 3.2. Calculation Results Analysis

**Figure 8:**
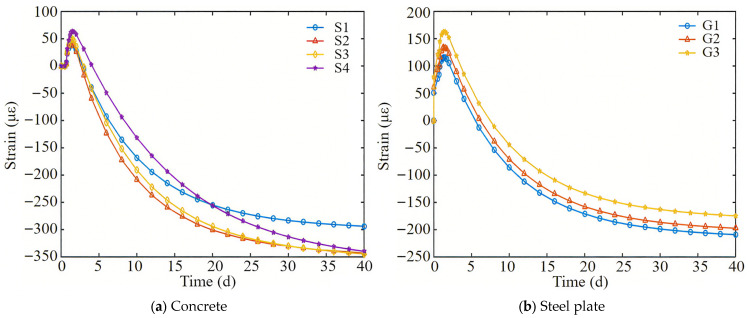
The calculated strain variation diagram of the full-section casting segment with time.

Figure 8a shows the variation in calculated strain over time for the full-section casting segment, corresponding to measurement points (S1–S4) on Section 1. From Figure 8a, it can be seen that in the early stage of concrete casting, the transverse strain of the segment increases. At this time, the hydration heat temperature of the concrete increases, causing the segment to expand. However, at this time, the concrete is in a flow plastic state, and the expansion amount is not large, with a maximum strain of 65 με. As time progresses, the strain gradually decreases. After 8 days of concrete casting, the transverse strains of the full-section casting segment can reach 151 με. The shrinkage strain of the bottom plate is smaller than that of the top plate, which is consistent with the measured strain. And the final transverse strain of the segment can reach −348 με.

From the previous analysis, it can be seen that the strain of concrete is mainly caused by the temperature drop of hydration heat and dry shrinkage. As shown in Figure 4 and Figure 5, when the concrete is poured at 5.5 days, the temperature is 39.3 °C and the strain is −110 με. When the temperature of the concrete decreases to 30 °C, the coefficient of thermal expansion of the concrete remains constant, and the temperature drop increases the strain to −203 με. Therefore, the strain caused by temperature drop and drying shrinkage of concrete accounts for 59% and 41% of the total strain, respectively. The temperature drop effect occurs in the early stage, while the drying shrinkage effect occurs in the later stage.

Figure 8b shows the strain diagram of the bottom steel plate calculation. The calculation point G1 in the figure corresponds to the measurement point G1 of the bottom steel plate. The calculation points G2 and G3 are located along the longitudinal direction of the segment, 1 m and 2.5 m away from point G1, respectively. According to Figure 8b, the bottom steel plate point G1 experienced a lateral expansion of 120 με in the early stage, and a shrinkage of 50 με after 8 days of concrete pouring, which is consistent with the measured values. Comparing points G1, G2, and G3, the further away from the post-casting belt, the greater the initial expansion of the bottom steel plate.

## 4. Analysis of Constraint Effect of the Post-Casting Belt

### 4.1. Stress Analysis of the Full-Section Casting Segment Without Constraints

**Figure 9:**
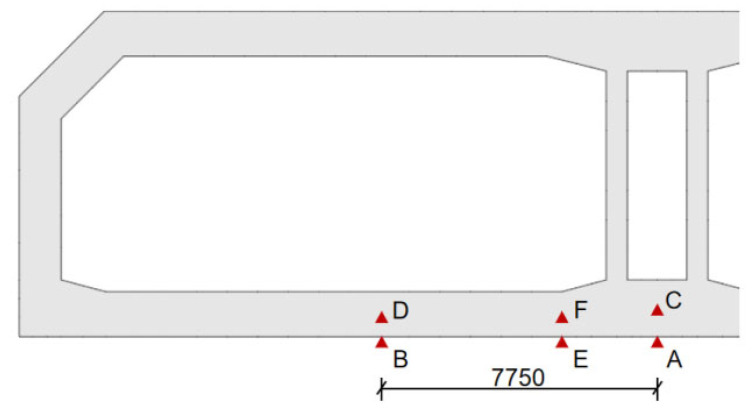
The layout diagram of calculation points [18].

For the convenience of analysis, six calculation points are selected at the section connected to the post-casting belt. Points A and C are located on the lower surface and center of the bottom plate at the center of the section, points B and D are located on the surface and center of the bottom plate in the middle of the driving lane, 7.75 m away from point A (point C), and points E and F are located on the surface and center of the bottom plate at the chamfer, as shown in Figure 9.

**Figure 10:**
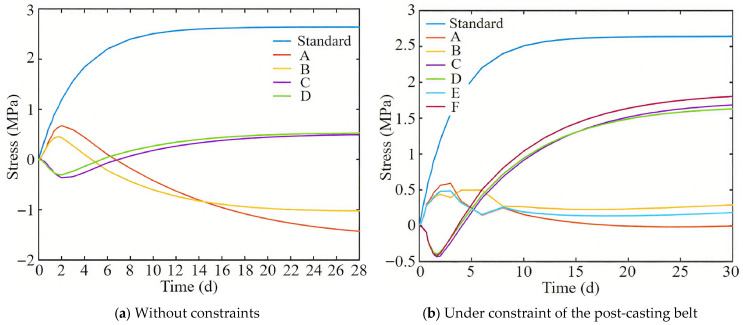
Stress variation diagram of the full-section casting segment with time.

Figure 10a shows the variation in stress at calculation points in the full-section casting segment without constraints over time. The diagram shows the allowable stress variation over time according to the Chinese “Standard for construction of mass concrete (GB50496-2018)” code [22], which is used to evaluate whether there is a risk of cracking in the immersed tube. From Figure 10a, it can be seen that the surface of the immersed tube (points A and B) is first subjected to tension and then to compression, while the center position (points C and D) is first subjected to compression and then to tension. This is due to the internal and external temperature difference, and the final hydration heat temperature field reserves temperature stresses of 0.5 MPa and −1.4 MPa at the center and surface of the immersed tube bottom plate, respectively.

### 4.2. Stress Analysis of the Full-Section Casting Segment Under Constraint of Post-Casting Belt

Figure 10b shows the variation in stress at calculation points in the full-section casting segment under the constraint of the post-casting belt over time. In the early stage of concrete pouring, the internal and external temperature difference in the concrete plays a dominant role, causing the surface position of the segment to be under tension and the center position to be under compression. The bottom plate is subjected to tension due to the constraint of steel bars and the bottom steel plate, and the tensile stress gradually increases under the combined action of temperature drop and post-casting belt constraint. The final tensile stresses at the center and surface are 1.8 MPa and 0.3 MPa, respectively.

Comparing Figure 10a,b, it can be seen that the steel plate and steel bars constraint of the post-casting belt greatly increase the tensile stress of the immersed tube bottom plate. Comparing the calculation points, the tensile stress increases by about 1.4 MPa.

**Figure 11:**
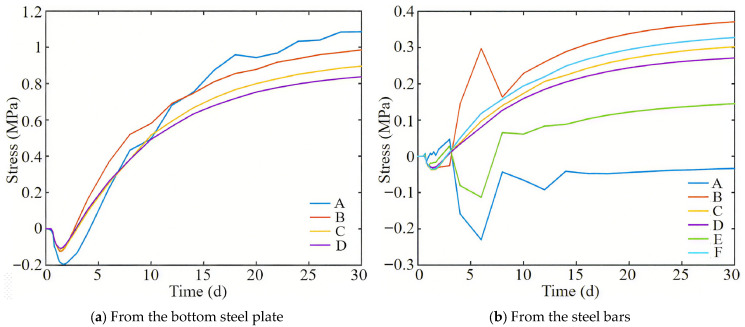
Constraint stress variation diagram of the full-section casting segment.

Figure 11 shows the influence of the bottom steel plate and steel bars of the post-casting belt on the stress of the full section casting segment bottom plate, respectively. It can be seen from Figure 11 that when the post-casting belt is 1.6 m long, the influence of the bottom steel plate and steel bars on the stress of the segment bottom plate is about 1 MPa and 0.4 MPa, respectively. Based on comparative analysis of on-site monitoring data, the constraint stress caused by the bottom steel plate is about 70% and the steel bars are about 30%.

### 4.3. Analysis of the Requirements for Setting the Length of the Post-Casting Belt

The above analysis shows that the constraint of the post-casting belt has an impact on the stress of the full-section casting segment. Further research will be conducted on the effect of the post-casting belt length on the constraint stress.

**Figure 12:**
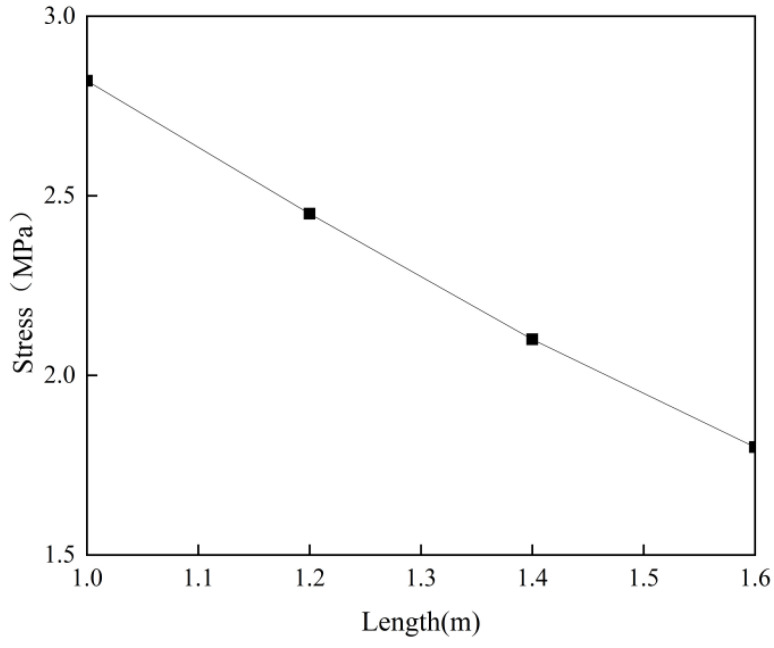
Stress variation diagram with post-casting belt length for full-section casting segments.

Figure 12 shows the relationship between the post-casting belt length and the stress of the full-section casting segment. The figure indicates that a shorter post-casting belt increases the constraint effect, leading to higher stress. To avoid concrete cracking, the post-casting belt length should not be less than 1.2 m, as per the allowable stress in the Standard. This is the primary reason for selecting a 1.6 m post-casting belt for the Chebei Tunnel.

The stress generated by the constraint of the post-casting belt on the full-section casting segment can be calculated according to Formula (7), where *β* is the constraint coefficient of the post-casting belt on the full-section casting segment.

(12) $$\sigma =\beta \cdot E_{c}\cdot \varepsilon_{tot}$$

**Figure 13:**
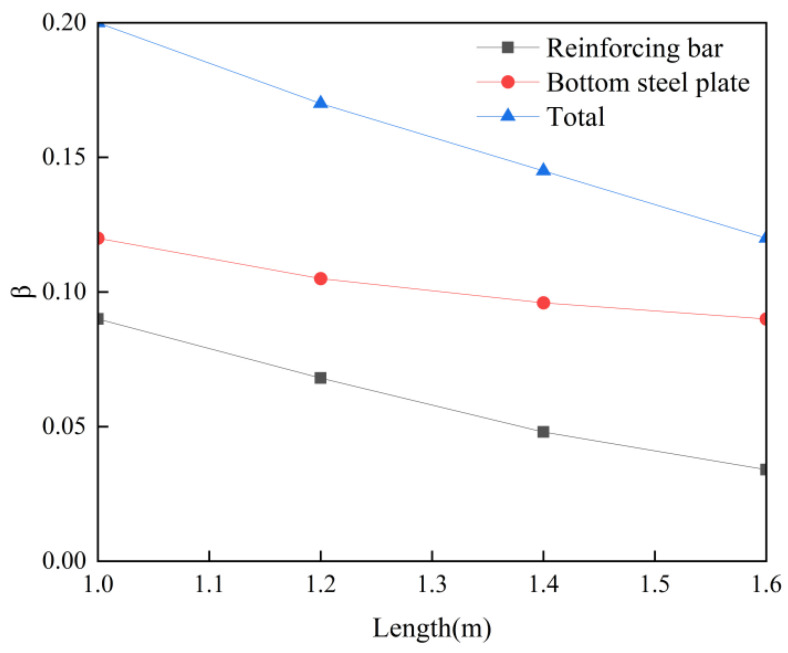
Constraint coefficients variation diagram with post-casting belt length.

By comparing Figure 8, Figure 9 and Figure 10 with Figure 11, it can be concluded that when the length of the post-casting belt is 1.6 m, the post-casting belt, the bottom steel plate, and the steel bars constraint coefficients are 0.12, 0.09, and 0.034, respectively. Further analysis of the constraint coefficients for different post-casting belt lengths is shown in Figure 13.

As shown in Figure 13, as the length of the post-casting belt increases, the post-casting belt constraint coefficient gradually decreases, and the rate of reduction in the steel bars constraint coefficient is faster than that of the bottom steel plate. In practical cases, the length of the post-casting belt is generally set to around 1.5 m, and the post-casting belt constraint coefficient is about 0.13.

## 5. Conclusions

This article compares and analyzes the constraint effects of the bottom steel plate and steel bars of the post-casting belt on the full-section casting segment through on-site testing and numerical calculation. Based on the requirements of concrete crack control, the setting requirements for the post-casting belt length are proposed. The main conclusions are as follows:

(1) Due to the combined effects of hydration, heat temperature field, and concrete shrinkage, the full-section casting segment first expands and then shrinks, with a final strain of 348 με. The strain caused by concrete temperature drop and shrinkage accounts for 59% and 41%, respectively.
(2) When the hydration heat temperature rises, due to the plastic flow state of the concrete, the expansion of the bottom steel plate is greater than that of the concrete. Subsequently, as the strength of the concrete increases, the bottom steel plate and the concrete form a force-bearing whole and shrink together with the temperature drop.
(3) This article proposes a method for determining the thermal expansion coefficient during the concrete pouring process through on-site testing and analysis, and verifies its reliability through numerical calculation and comparative analysis. However, due to limited monitoring data, we will verify the reliability of the calculation formula through data from multiple projects in the future.
(4) The post-casting belt exerts a restraining effect on the full-section casting segment, and the shorter the post-casting belt, the greater the restraining effect. The post-casting belt length of the Chebei Tunnel is 1.6 m, and the post-casting belt restraint causes an increase of 1.4 MPa in the tensile stress of the full-section casting segment. Based on comparative analysis of on-site monitoring data, the contribution of the bottom steel plate and steel bars restraint accounts for about 70% and 30%, respectively. And as the post-casting belt length becomes shorter, the contribution of steel bars will increase.
(5) In practical engineering, the post-casting belt is often set at around 1.5 m, and the constraint coefficient of the post-casting belt can be taken as 0.13.

## Figures and Tables

**Table 1:** Concrete mix proportion of the Chebei project.

Binder Content (kg/m^3^)	Water-to-Binder Ratio	Cement (kg/m^3^)	Fly Ash (kg/m^3^)	Slag Powder (kg/m^3^)	Sand Ratio	Water Reducer Content	Density (kg/m^3^)
400	0.39	210	80	110	40%	1.2%	2360

## Data Availability

The original contributions presented in this study are included in the article. Further inquiries can be directed to the corresponding author.
